# Knowledge and associated factors towards cytotoxic drug handling among University of Gondar Comprehensive Specialized Hospital health professionals, institutional-based cross-sectional study

**DOI:** 10.1186/s12199-020-00850-z

**Published:** 2020-04-13

**Authors:** Wudneh Simegn, Baye Dagnew, Henok Dagne

**Affiliations:** 1 Department of Pharmaceutics, School of Pharmacy, University of Gondar, P.O.Box 196, Gondar, Ethiopia; 2 Department of Human Physiology, School of Medicine, University of Gondar, P.O.Box 196, Gondar, Ethiopia; 3 Department of Environmental and Occupational Health and Safety, Institute of Public Health, University of Gondar, P.O.Box 196, Gondar, Ethiopia

**Keywords:** Cytotoxic drugs, Knowledge, Health professional

## Abstract

**Background:**

Currently, cancer is among the leading causes of morbidity and mortality in the world. Exposure to CDs may occur during drug preparation and mixing, during drug administration, during transport, and cleaning spills and waste disposal. Healthcare workers who prepare or administer antineoplastic drugs, or who work in areas where these drugs are used, can be exposed to these agents. This also affects the public around the exposed area if appropriate disposal system is not known. Several studies reported increased risks of leukemia and breast cancer among nurses handling CDs and not following safety guidelines. Because of the absence of studies in Ethiopia, the current study was conducted to determine the knowledge level of cytotoxic drug handling and associated factors among health professionals in the University of Gondar Comprehensive Specialized Hospital.

**Methods:**

The institutional-based cross-sectional study was conducted from June to August 2019. Epi info 7.1 was used for data entry and then exported into SPSS version 20 for computing, recording, and statistical analysis. Logistic regression was used to explain the relationship with independent variables.

**Results:**

Four hundred and twelve health professionals participated in the study with 53.4% males. The participants’ mean age was 29.9 (± 5.43) years ranging from 20–60. Two hundred and twenty-three (54.1%) health professionals heard about cytotoxic drugs, and 52.7% (95% UI 47.8–57.8%) had good knowledge of cytotoxic drug handling. Being male sex (AOR = 1.84, 95% CI (1.13–3.00)), age of 29–31 (AOR = 1.99, 95% CI (1.03–3.84)), hearing information about cytotoxic drug handling (AOR = 2.53, 95% CI (1.43–4.47)), ever attended training on cytotoxic drug handling (AOR = 3.15, 95% CI (1.13–8.79)), ever taking courses related to cytotoxic drugs (AOR = 2.03, 95% CI (1.15–3.59)), and good practice (AOR = 3.24, 95% CI (1.95–5.37)) were significantly associated with knowledge towards cytotoxic drug handling. It is therefore imperative to train health professionals and to incorporate CDs handling related course contents while revising curricula to raise the knowledge of health professionals about proper cytotoxic drug handling.

**Conclusion:**

Above half of the study participants scored higher than the median of the cytotoxic drug handling knowledge questions. Sex, age, information about cytotoxic drug handling, training, taking courses related to cytotoxic drugs, and good practice were significantly associated with knowledge towards cytotoxic drugs handling.

## Background

Currently, cancer is among the leading causes of morbidity and mortality in the world. Chemotherapeutic agents are cytotoxic drugs (CDs) which are extensively practiced in health care facilities to treat cancer [1]. Their pharmacological property to kill tumor cells is by interfering with cell division. However, their action is not specific to cancerous cells, and non-cancerous cells may also get damaged [2].

Exposure to CDs may occur during drug preparation and mixing [1, 3], during drug administration [4], during transport, and cleaning spills and waste disposal [5]. Significant amounts of CDs can be absorbed via inhalation of the powder and liquid aerosols, unprotected skin, and mucous membranes; oral exposure may occur from hand-to-mouth contact or ingestion of or drinks and (iv) needle stick injury [6–8].

Healthcare workers who prepare or administer antineoplastic drugs, or who work in areas where these drugs are used, can be exposed to these agents when they are present on contaminated work surfaces, drug vials and containers, contaminated clothing and medical equipment, and patient excreta and secretions such as urine, feces, and sweat [9]. Several studies reported increased risks of leukemia and breast cancer among nurses handling CDs and not following safety guidelines [10].

Several studies revealed that CDs are hazardous to healthcare workers particularly nurses, pharmacists, and cleaners who may come in contact with these CDs during their daily work activities [11]. The number of healthcare workers who are in contact with cytotoxic drugs is expected to increase due to the increase in the number of new cancer cases requiring chemotherapy [12].

Cytotoxicity can be due to direct contact with CDs without personal protective equipment, needle stick injury, spills, and other unintended exposures which can be contributed by lack of training, inadequate controls, and poor communication [13]. Although guidelines for safe handling of CDs were introduced more than 20 years ago, contamination of both the working environment as well as the healthcare workers is still reported in several recent studies mainly in developing countries [9, 14]. Chronic health effects of cytotoxicity include mutation, carcinogenicity, and adverse reproductive outcomes like infertility, abortion, and poor neonatal outcomes [15–17].

The cytotoxicity of antineoplastic drugs is preventable using different mechanisms including the use of personal protective equipment and understanding safety guidelines. Acquiring such ways is through the acquisition of knowledge in training or taking the course [18]. Previous studies regarding knowledge revealed a need for training and delivering protective equipment to those working in oncology units [19, 20]. Knowledge about cytotoxic drug handing can help professionals in reducing cytotoxicity of CDs which is associated with the job rank of professionals [21]. Antineoplastic drugs (CDS) are known to cause problems to health workers, the environment, and the general public [22]. CDS can induce significant side effects in patients or any other person exposed to them [23]. CDS, if not properly handled, may end up in the drinking water and put the general public to several health consequences [24]. The characteristic feature of each health-care waste is to pose risk to patients, staff, and the environment [25]. Although guidelines for safe handling of CDs were introduced earlier, contamination of both the working environment as well as the healthcare workers is still reported in previous studies particularly in developing countries [9, 14]. Lack of knowledge among other factors was a major determinant of unsafe behavior related to handling of CDs by healthcare workers [9, 26].

Medical staff and administration have to be more attentive and responsible in the collection and disposal of waste. The knowledge regarding CDs among health professionals is important to safeguard themselves, the patients, and the general public through safe operating procedures and public education about safe disposal of unused drugs.

Because of the aforementioned problems related to cytotoxic drugs, the relevance of healthcare professional’s knowledge about CD handling and absence of studies in Ethiopia, the authors conducted this study to put baseline results for patients, health sector stakeholders, health professionals, and public health safety. The current study assessed the level of knowledge of health professionals who were working in the University of Gondar Comprehensive Specialized Hospital.

## Methods

### Study setting, design, and period

This institutional-based cross-sectional study was conducted in the University of Gondar Comprehensive Specialized Hospital, northwest Ethiopia, which is located 728 km away from Addis Ababa, the capital city of Ethiopia. The actual data collection period was from June to August 2019.

### Source population

All the University of Gondar Comprehensive Specialized Hospital health professionals were working in different departments of the hospital including cancer treatment center.

### Study population

All the University of Gondar Comprehensive Specialized Hospital health professionals were working in different departments of the hospital including cancer treatment center and were present at the time of data collection from June to August 2019.

### Inclusion and exclusion criteria

All health professionals working in the University of Gondar Comprehensive Specialized Hospital were included. Health professionals who were severely ill during the data collection period and those health professionals who had confirmed cancer were excluded.

### Sample size calculation and sampling technique

The sample size (*n*) was calculated using single population proportion formula with assumptions of the proportion = 0.5 (no previous study in Ethiopia), 95% uncertainty interval, and margin of error (*d*) = 5%. After adding a non-response rate of 10%, the final sample size was 423. Simple random sampling technique was used for the selection of health professionals to be included in the study.

### Data collection procedure

A semi-structured, pretested self-administered questionnaire was used to collect the required data. It consisted of different items regarding sociodemographic characteristics, knowledge, attitude, and practice on cytotoxic drug handling. The cytotoxic drug handling related questions were adapted from another study [27]. Four BSc Nurses were recruited to distribute and return the questionnaire.

### Variables of the study

#### Dependent variable

It is a knowledge to cytotoxic drug handling.

#### Independent variables

The following are the independent variables: sex, age in years, marital status, educational level, work experience, working setting, profession, use of personal protective equipment, working hours per week, work stress, heard about cytotoxic drugs, ever attended training, ever working in cancer center, ever taking courses related to CD, availability of personal protective equipment, and practice and attitude towards cytotoxic drug handling.

### Operational definition

#### Knowledge

Respondents were asked 13 knowledge questions about whether they knew of anti-cancer drugs are cytotoxic, routes of exposure to CDs, adverse health effects of exposure to CDs, management of adverse health effects of CDs, guidelines and standards for safe preparation of CDs, safe administration of CDs, safe transport and storage of CDs, use of biological safety cabinet for all preparations, management of accidents in handling of CDs, required personal protective equipment (PPE), how to use PPE correctly, and safe CD waste disposal methods. Respondents were awarded 1 point for each right answer and 0 for wrong reply. The sum was dichotomized as good and poor using median score as the data was not normally distributed. The mean score was 4.91, and the median score was 4.0. Study participants who scored median and above of the questions were considered as having good knowledge.

#### Practice

Respondents were asked 12 practice questions about whether they always prepare CDs in preparation room, always prepare CDs in biological safety cabinet, never do risky behaviors like eat and drink in preparation room, do not store CDs in preparation room, follow guidelines and standards for handling of CDs, always wear personal protective equipment during preparations of CDs, always wear personal protective equipment during administration of CDs, use biological safety cabinet for all preparations, manage accidents in handling of CDs, always wear personal protective equipment during transport and storage of CDs, manage accidents as spoils based on standard protocols, record and report all accidents in handling of CDs, consult clinical pharmacist about safe handling of CDs, and consult occupational medicine specialists about related health problems. Respondents were given 0 and 1 point for each inappropriate and appropriate CD handling practice reported. The mean score of the practice questions was 4. Study subjects who scored 6 to 12 out of the 12 practice questions (≥ 50%) were considered as having good practice.

#### Cytotoxic

It is a substance or agent synonymous with antineoplastic agents and anticancer agents.

#### Handling

It involves receiving, processing, planning and compounding, administration, and cleaning and disposal.

### Statistical analysis

Epi info 7.1 was used for data entry and then exported into SPSS version 20 for computing, recording, and statistical analysis. Range, mean with standard deviation (SD), frequency, and percent were computed to articulate the descriptive results of the study. Logistic regression was used to explain the relationship between knowledge about cytotoxic drug handling and independent variables. The bivariable analysis was executed to determine the crude association between knowledge and each independent variables. Independent variables with a *p* value of < 0.2 were selected for multivariable logistic regression. A variable with a *p* value of ≤ 0.05 with 95% uncertainty interval was treated as a significant factor for knowledge towards cytotoxic drug handling.

### Data quality control

Quality control was considered starting from questionnaire design until the analysis process. It was pretested, and data collection facilitators were trained about the purpose of the study and ethical issues in the process of data filling.

## Results

Four hundred and twelve health professionals participated in the study with a response rate of 97.4%. Two hundred and twenty (53.4%) participants were males with a mean age of 29.9 years ranging from 20 to 60 years old. Three hundred and thirty-nine (82.3%) participants attain an educational level of bachelor’s degree, 142 (36.5%) had 2 years work experiences, and only 27 (6.6%) individuals had working experiences in oncology unit with 13 (3.2%) workers working in cancer center currently (Table 1).

**Table 1:** Sociodemographic characteristics of study participants in the University of Gondar Comprehensive Specialized Hospital, Gondar, Ethiopia, 2019 (*n* = 412)

Variable	Categories	Frequency	Percent
Marital status	Married	226	54.9
	Unmarried	186	45.1
Sex	Female	192	46.6
	Male	220	53.4
Age in years	Mean = 29.92 (± 5.43), Min = 20, Max = 60		
Education level	Diploma	26	6.3
	Bachelor degree	339	82.3
	Masters and above	47	11.4
Work experience in years	2	142	36.5
	3–4	66	16.0
	5–8	137	33.3
	8–37	67	16.3
Ever working in cancer center	Yes	42	10.2
	No	370	89.8
Profession	Nurse	196	47.6
	Pharmacy	112	27.2
	Medicine	18	4.4
	Midwifery	69	16.7
	Laboratory	17	4.1
Current work setting	Cancer center	13	3.2
	Others	399	96.8
Use of personal protective equipment	Yes	121	29.4
	No	291	70.6
Working hours per week	30–38	45	10.9
	39–43	161	39.1
	44–55	97	23.5
	56–110	109	26.5
The average number of patients contacted per day	4–9	100	24.3
	10–14	85	20.6
	15–29	59	14.3
	30–200	168	40.8
Workload	None	32	7.8
	Low	43	10.4
	Medium	165	40.0
	High	172	41.7

### Knowledge towards cytotoxic drugs

Two hundred and twenty-three (54.1%) participants heard about cytotoxic drugs. Only 10% of participants attended training about cytotoxic drugs with 5.6% health professionals attended training in the last 2 years, and 41.3% of professionals took related courses about cytotoxic drugs. From the participants, 52.7% (95% UI 47.8–57.8%) had a good knowledge regarding cytotoxic drug handling (Table 2).

**Table 2:** Items related to knowledge towards cytotoxic drugs among health professionals at the University of Gondar Comprehensive Specialized Hospital, Northwest Ethiopia, 2019 (*n* = 412)

Variable	Categories	Frequency	Percent
Heard about cytotoxic drug handling	Yes	223	54.1
	No	189	45.9
Ever attended training	Yes	41	10.0
	No	371	90.0
Taking training in 2 years	Yes	23	5.6
	No	389	94.4
Ever worked in oncology department	Yes	42	10.2
	No	370	89.8
Ever taking a course directly related with cytotoxic drug handling	Safe preparation	53	12.9
	Safe administration	50	12.1
	Safe transport	40	9.7
	General oncology	19	4.6
	Chemotherapy	118	28.6
	Radiotherapy	19	4.6
	Hazards of CDS	16	3.9
Presence of cytotoxic drug handling manual on working setting	Yes	82	19.9
	No	330	80.1
Knowledge towards CD handling	Good	217	52.7
	Poor	195	47.3
Attitude	Good	224	54.4
	Poor	188	45.6
Practice	Good	155	37.6
	Poor	257	62.4

### Determinant factors of knowledge towards cytotoxic drugs

Sex, age, marital status, educational level, working experience in oncology, department, working in cancer center, heard about cytotoxic drug handling, ever attending training on cytotoxic drugs, ever working in oncology unit, ever taking courses related to cytotoxic drugs, availability of manual, availability of PPE, and desirable practice towards cytotoxic drugs were candidate variables for multivariable logistic regression. In the final model, male sex (AOR = 1.84, 95% CI (1.13–3.00)), age of 29–31 (AOR = 1.99, 95% CI (1.03–3.84)), hearing about cytotoxic drug handling (AOR = 2.53, 95% CI (1.43–4.47)), ever attended training on cytotoxic drug handling (AOR = 3.15, 95% CI (1.13–8.79)), ever taking courses related to cytotoxic drugs (AOR = 2.03, 95% CI (1.15–3.59)), and good practice (AOR = 3.24, 95% CI (1.95–5.37)) were significantly associated with knowledge towards cytotoxic drugs (Table 3).

**Table 3:** Associated factors of knowledge towards cytotoxic drugs handling among health professionals at the University of Gondar Comprehensive Specialized Hospital, Gondar, Ethiopia, 2019 (*n* = 412)

Variables	Categories	Knowledge		COR (95% CI)	AOR (95% CI)
		Good (%)	Poor (%)		
Sex	Male	129 (58.6)	91 (41.4)	1.68 (1.13,2.48)	1.84 (1.13,3.00)*
	Female	88 (45.8)	104 (54.2)	1	1
Age in years	20–25	32 (46.4)	37 (53.6)	0.82 (0.45,1.49)	1.35 (0.62,2.93)
	26–28	68 (51.1)	65 (48.9)	1.00 (0.61,1.63)	1.32 (0.70,2.46)
	29–31	56 (61.5)	35 (38.5)	1.52 (0.87,2.65)	1.99 (1.03,3.84)*
	32–60	61 (51.3)	58 (48.7)	1	1
Marital status	Married	121 (53.5)	105 (46.5)	1.08 (0.73,1.59)	1.48 (0.88,2.48)
	Unmarried	96 (51.6)	90 (48.4)	1	1
Education level	Diploma	17 (65.4)	9 (34.6)	1	1
	BSc degree	178 (52.5)	161 (47.5)	0.59 (0.25,1.35)	2.72 (0.79,9.42)
	Masters +	22 (46.8)	25 (53.2)	0.47 (0.17,1.25)	1.30 (0.62,2.77)
Ever working in cancer center	Yes	20 (74.1)	7 (25.9)	2.73 (1.13,6.60)	1.66 (0.42,6.55)
	No	197 (51.2)	188 (48.8)	1	1
Profession	Nurse	94 (48.0)	102 (52.0)	1.69 (0.60,4.75)	0.93 (0.29,3.02)
	Pharmacy	76 (67.9)	36 (32.1)	3.87 (1.33,11.23)	1.46 (0.42,5.03)
	Medicine	11 (61.1)	7 (38.9)	2.88 (0.73,11.38)	1.19 (0.24,6.03)
	Midwifery	30 (43.5)	39 (56.5)	1.41 (0.47,4.27)	0.89 (0.26,3.06)
	Laboratory	6 (35.3)	11 (64.7)	1	1
Current work setting	Cancer center	11 (84.6)	2 (15.4)	5.15 (1.13,23.55)	3.77 (0.49,28.80)
	Others	206 (51.6)	193 (48.4)	1	1
Heard about CD handling	Yes	149 (66.8)	74 (33.2)	3.58 (2.38,5.38)	2.53 (1.43,4.47)***
	No	68 (36.0)	121 (64.0)	1	1
Ever attending training on CD handling	Yes	35 (85.4)	6 (14.6)	6.06 (2.49,14.75)	3.15 (1.13,8.79)*
	No	182 (49.1)	189 (50.9)	1	1
Ever working in oncology department	Yes	32 (76.2)	10 (23.8)	3.2 (1.53,6.70)	1.48 (0.57,3.89)
	No	185 (50)	185 (50)	1	1
Ever taken courses related to CDs	Yes	117 (68.8)	53 (31.2)	3.14 (2.07,4.74)	2.03 (1.15,3.59)*
	No	100 (41.3)	142 (58.7)	1	1
Presence of cytotoxic drug handling manual on working setting	Yes	53 (64.6)	29 (35.4)	1.85 (1.12,3.06)	1.41 (0.70,2.84)
	No	164 (49.7)	166 (50.3)	1	1
Availability of PPE	Yes	74 (61.2)	47 (38.8)	1.63 (1.06,2.51)	1.32 (0.75,2.31)
	No	143 (49.1)	148 (50.9)	1	1
Practice	Good	105 (67.7)	50 (32.3)	2.72 (1.79,4.13)	3.24 (1.95,5.37)***
	Poor	112 (43.6)	145 (56.4)	1	1

Hosmer and Lemeshow god-ness of fit *p* = 0.476, *p < 0.05, and ***p < 0.001

*PPE* personal protective equipment

## Discussion

This study aimed at assessing the knowledge and contributing factors of health professionals about CD handling. Long-term occupational exposure to cytotoxic drugs is associated with various carcinogenic, teratogenic, and mutagenic effects [17, 28, 29]. High levels of knowledge on cytotoxic drugs and their adverse health effects are essential to improving adherence by health professionals with safety measures [9]. Health professionals’ knowledge of CDs is vital for safeguarding themselves, the patients, and the general public through safe operating procedures and public education about proper disposal of unused medications. Lack of knowledge was one of the major determinants of unsafe behavior related to handling of CDs by healthcare workers as evidenced from previous studies [9, 26].

In the current study, 52.7% (47.8–57.8%) of the health professionals had good knowledge about cytotoxic drug handling. The proportion of health professionals with good knowledge about CDs handling in the current study was lower than previous reports among healthcare professionals in Kenya [27], nurses in Turkey [30], nurses and pharmacists in Italy [31], and oncology nurses in Egypt [23]. The lower proportion of knowledge about CDs in the current study might be because cancer treatment units are recently established in the university. The other reason might be the difference between the study setting and the study participants included.

In this study, sociodemographic factors such as male sex, age of 29 to 31 years, having information about CD and attending training or courses related to CDs handling, and good CD handling practice were associated with knowledge about CDs handling.

Male health workers had better odds of knowledge as compared to females. This is in line with a few previous studies [20, 32]. However, a study in Saudi Arabia revealed significantly higher knowledge among females [33]. These discrepancies might be due to other latent factors that mediate the knowledge than sex disparity.

Respondents aged 29–31 years were more likely to have better knowledge regarding cytotoxic drug handling. However, age was not significantly associated with knowledge of CDs handling in previous studies [34]. Study participants who ever heard about proper CD handling were more likely to have better CDs handling knowledge. This is not surprising as knowledge is acquired through information. Health professionals who received training about CD handling were more likely to have better CD handling knowledge as compared to those who did not attend training. Several previous studies supported that training improves knowledge about cytotoxic drug handling [9, 23, 29, 35–38].

Finally, participants who had taken courses directly related to CDs handling were more likely to have better knowledge regarding CDs, and those with good self-reported CDs handling practice were more than 3 times more likely to have good knowledge. This might be because a high level of knowledge will enable for safe CDs handling compliance, and the practice leads to the acquisition of knowledge. The association of practice with knowledge was in line with a previous study [9]. Nonetheless, the practice has no association with knowledge in other studies [23, 27].

Some of the limitations of this study include social desirability bias and recall bias. The use of relative scale than global sum score in analyzing the response variable in the current study made the comparison with earlier studies difficult. The generalizability of the result to other countries or regions might not be possible as the socio-cultural context, and the health service delivery is different from country to country. It is not known whether similar results will be obtained in other countries and/or regions. Researchers might be expected to adapt the scale used to assess the cytotoxic drug handling knowledge to their own context. Besides, the cause-effect relationship cannot be established as this is a cross-sectional study conducted at a medical hospital in Ethiopia.

## Conclusion

Above half of the study participants scored higher than the median of the cytotoxic drug handling knowledge questions. Sex, age, information about cytotoxic drug handling, training, taking courses related to cytotoxic drugs, and good practice were significantly associated with knowledge towards cytotoxic drugs handling. It is therefore imperative to train health professionals and to incorporate CDs handling related course contents while revising curricula to raise the knowledge of health professionals about appropriate cytotoxic drug handling.

## Data Availability

The dataset is accessible at the corresponding author upon reasonable request.

## Abbreviations

AOR: Adjusted odds **r**atio
CD: Cytotoxic drug
COR: Crude odds ratio
EPI info: Epidemiological information
SPSS: Statistical Package for Social Sciences
UI: Uncertainty interval
